# HM71224, a selective Bruton’s tyrosine kinase inhibitor, attenuates the development of murine lupus

**DOI:** 10.1186/s13075-017-1402-1

**Published:** 2017-09-26

**Authors:** Yu-Yon Kim, Ki Tae Park, Sun Young Jang, Kyu Hang Lee, Joo-Yun Byun, Kwee Hyun Suh, Young-Mi Lee, Young Hoon Kim, Kwang Woo Hwang

**Affiliations:** 1 Host Defense Modulation Lab, College of Pharmacy, Chung-Ang University, 84 Heukseok-Ro, Dongjak-Gu, Seoul 06974 Republic of Korea; 2 Hanmi Research Center, Hanmi Pharm. Co. Ltd, 550 Dongtangiheung-Ro, Hwaseong-Si, Gyeonggi-Do 18469 Republic of Korea

## Abstract

**Background:**

Systemic lupus erythematosus (SLE) is associated with B cell hyperactivity, and lupus nephritis (LN), in particular, is promoted by the production of autoantibodies and immune complex deposition. Bruton’s tyrosine kinase (BTK) plays critical roles in B cell receptor-related and Fc receptor-related signaling. We aimed to investigate the impact of therapeutic intervention with HM71224 (LY3337641), a selective BTK inhibitor, on the development of murine SLE-like disease features.

**Methods:**

We examined the therapeutic effects of HM71224 on SLE-like disease features in MRL/*lpr* and NZB/W F1 mice. The disease-related skin lesion was macroscopically observed in MRL/*lpr* mice, and the impact on splenomegaly and lymphadenopathy was determined by the weight of the spleen and cervical lymph node. The renal function was evaluated by measuring blood urea nitrogen, serum creatinine, and urine protein, and the renal damage was assessed by histopathological grading. Survival rate was observed during the administration period. The impact of B cell inhibition was investigated in splenocytes from both mice using flow cytometry. Autoantibody was measured in serum by ELISA.

**Results:**

HM71224 effectively suppressed splenic B220^+^GL7^+^, B220^+^CD138^+^, and B220^+^CD69^+^ B cell counts, and anti-dsDNA IgG and reduced splenomegaly and lymph node enlargement. The compound also prevented skin lesions caused by lupus development, ameliorated renal inflammation and damage with increased blood urea nitrogen and creatinine, and decreased proteinuria. Furthermore, HM71224 also decreased mortality from lupus development in both mouse models.

**Conclusion:**

Our results indicate that inhibition of BTK by HM71224 effectively reduced B cell hyperactivity and significantly attenuated the development of SLE and LN in rodent SLE models.

## Background

Systemic lupus erythematosus (SLE) is a chronic inflammatory autoimmune disease [1, 2], and lupus nephritis (LN) is one of the most severe SLE-associated organ complications [3, 4]. The etiology of SLE has been still unclear, however, abnormalities of genetic variants, that of molecular pathways, and dysregulation of several immune cells have been previously reported as disease pathogenesis in SLE. Dysregulation of B cell receptor (BCR) signaling that can lead to autoimmunity including autoantigen presentation to activate T cells and pro-inflammatory signaling by cytokines secretion and complement activation have been implicated in pathogenesis of SLE and LN [5, 6]. Especially, several pathogenic autoantibodies secreted by abnormalities of B cells play crucial roles in pathogenesis of SLE. Moreover, autoantibodies and intrarenal complement activation contribute to the renal immune-pathogenesis of LN [7–9]. Given the crucial role of B cells in SLE pathogenesis, they have emerged as promising new targets for SLE and LN treatment in the last decade. Clinical trials of several B cell-targeted biological treatments, such as the anti-CD20 antibody rituximab [10–12], B cell-activating factor inhibitor belimumab [13], and anti-CD22 antibody epratuzumab [14, 15], have been conducted, but only belimumab has demonstrated a clinical benefit, leading to US Food and Drug Administration approval [16, 17].

Bruton’s tyrosine kinase (BTK) is a non-receptor tyrosine kinase belonging to the tec protein tyrosine kinase (TEC) kinase family that is encoded by the *TEC* gene. BTK plays critical roles in activation mediated by the B cell receptor (BCR), Fc receptor (FcR), Toll-like receptor (TLR), and chemokine receptor [18–20]. In humans, BTK deficiency causes inherited X-linked agammaglobulinemia [21]. In mice, it leads to X-linked immunodeficiency (*xid*) [21] and BTK-knockout mice carrying the 56R anti-DNA Ig transgene do not produce anti-DNA antibodies [22]. Moreover, macrophages in BTK-deficient *xid* mice have impaired proinflammatory cytokine generation because BTK plays a major role in immune complex-mediated activation via FcγR in macrophages [21]. BTK is closely associated with SLE and LN development via both BCR and Fc receptor signaling, and therapeutic potential of BTK inhibitors have been demonstrated in several SLE animal models. For example, it have been reported that the therapeutic effects for glomerular nephritis of RN486 and PF-06250112, selective BTK inhibitors, by inhibition of effector cells and target autoantibodies in NZB/W F1 mice with Fc receptor dependent and antibody mediated LN [23, 24]. Ibrutinib also ameliorated humoral and cellular autoimmunity by partial crippling of cell signaling in both B cells and antigen presenting cells in lupus–prone B6.Sle1 and B6.Sle1.Sle3 mice [25]. Thus, targeting the BTK signaling pathway may provide an effective therapeutic strategy [26].

HM71224, orally active and irreversible small-molecule BTK inhibitor, may covalently bind to the active site (cysteine 481 residue) of BTK. HM71224 inhibited BTK, BMX, TEC and TXK which carry a conserved cysteine in the binding pocket in more than 85-kinase assays. HM71224 showed a highly selective inhibition for BTK with IC_50_ of 1.95 nM. The selectivity toward other BMX, TEC and TXK were 0.3, 2.3 and 2.4 fold, respectively. Moreover, HM71224 completely occupied to BTK and revealed potent inhibition of BCR, FcR and TLR mediated signaling [27]. 

In this study, to determine whether HM71224 could attenuate SLE and LN by suppressing BTK activation, the MRL/*lpr* and New Zealand Black/White F1 (NZB/W F1) mouse models, which have physiological relevance for human lupus, were used. MRL/*lpr* mice are homozygous for the lympho-proliferation spontaneous mutation (Fas^*lpr*^) and have impaired central tolerance [28, 29], and NZB/W F1 mice co-express several *sle* loci that are associated with increased risk of SLE development [30].

## Methods

### Immunoblotting

Ramos cells (American Type Culture Collection, Manassas, VA, USA), the human Burkitt’s lymphoma cell line, were cultured in suspension using Roswell Park Memorial Institute (RPMI) 1640 medium (Gibco, Rockville, MD, USA) with 10% fetal bovine serum (Gibco) at 37 °C in 5% CO_2_ in air. Cells were starved of serum for 1 h and then treated with HM71224 for 1 h. Cells were washed with phosphate-buffered saline (Welgene, Korea) and then were stimulated with 1 μg/ml of goat F(ab’)2 anti-human IgM (Southern Biotech, Birmingham, AL, USA) for 10 min on ice. Cells were then lysed in radioimmunoprecipitation assay (RIPA) buffer (Sigma-Aldrich, St. Louis, MO, USA) and the proteins were separated by SDS-PAGE (Bio-Rad Laboratories, Hercules, CA, USA) and transferred to polyvinylidene fluoride membranes (EMD Millipore, Billerica, MA, USA). Proteins were detected with anti-BTK Y223 (Cell Signaling Technology, Danvers, MA, USA), anti-BTK (53/BTK, Cell Signaling Technology), anti-PLCγ2 Y1217 (Cell Signaling Technology), anti-PLCγ2 (Cell Signaling Technology), and anti-GAPDH (Santa Cruz Biotechnology, Santa Cruz, CA, USA) antibodies. Protein signals were visualized by chemiluminescence using ECL western blotting detection reagent (EMD Millipore), and scanned blots were quantified using a Multi-Image analyzer (Fujifilm, Japan). The percentage of phosphorylation of each lane was determined using Multi-Gauge software (Fujifilm). The IC_50_ value was calculated using control IgM^+^ set at 100% and control IgM^-^ set at 0%.

### FcγR-stimulated cytokines

THP-1 cells (American Type Culture Collection) were differentiated to macrophages by stimulation with interferon-γ (IFN-γ) at 10 ng/ml in RPMI 1640 medium (Gibco) with 10% fetal bovine serum (Gibco) for 6 days at 37 °C in 5% CO_2_ in air. Monocyte-derived macrophages were pre-treated with HM71224 for 30 min. Cells were stimulated with a high concentration of plate-bound human IgG. The supernatant was collected after 24 h, and TNF-α and IL-6 were measured using commercially available ELISA kits (R&D Systems, Minneapolis, MN, USA).

### Experimental protocol for MRL/*lpr* mice and NZB/W F1 mouse models

Female MRL/MpJ-Fas *lpr*/J mice (20 ± 5 g, 8 weeks old) and female NZB/W F1 mice (30 ± 5 g, 18 weeks old) were obtained from SLC Inc. (Japan). Animals were housed and handled in a temperature-controlled environment with a 12-h light/12-h dark cycle. They had free access to standard pelleted food (Picolab Rodent diet 5053, St. Louis, MO, USA) and water *ad libitum*. In MRL/*lpr* mice, 12 animals per group identified by their urine protein score [31] were treated with HM71224 orally once per day from 8 weeks through 28 weeks of age, and in NZB/W F1 mice, 12 animals per group were administered HM71224 orally once daily from 18 weeks through 40 weeks of age. All animal experimental protocols and procedures were approved by the Institutional Animal Care and Use Committee of the Hanmi Research Center and performed according to the approved guideline.

### Skin lesion scores

Macroscopic lupus-erythematosus-like skin lesions were evaluated only in MRL/l*pr* mice with the spontaneous development of skin lesions similar to those seen in human SLE [32]. Skin lesions were scored every week and were expressed using a scoring system from 0 to 3 (0, none; 1, mild; 2, moderate (<2 cm); 3, severe (≥ 2 cm)) for changes in the nose, ears, and intrascapular region.

### Urine protein

Urine protein was individually evaluated weekly or biweekly using a urine test strip (URiSCAN strips, YD Diagnostics, Korea). Scores for protein concentration were graded from 0 to 4: 0, none; 1, 30–99 mg/dl; 2, 100–299 mg/dl; 3, 300–1999 mg/dl; 4, ≥ 2000 mg/dl or death.

### Blood urea nitrogen, creatinine, and autoantibody measurements

Blood samples were obtained from mice via the caudal vena cava under anesthesia with ether. Blood was centrifuged at 13,400 *g* for 2 min at 4 °C to collect serum. Blood urea nitrogen (BUN) and creatinine (CRE) were measured using a Hitachi 7020 automatic chemical analyzer (Hitachi, Japan). Serum mouse anti-dsDNA antibody levels were determined using a commercially available ELISA kit (Alpha Diagnostic, San Antonio, TX, USA) according to the manufacturer’s instructions.

### Kidney histopathological analysis

The kidneys were surgically removed under anesthesia with ether. They were fixed in 10% neutral buffered formalin and embedded in paraffin. Sections of 4-μm thickness were stained with hematoxylin and eosin and Periodic acid Schiff. The histopathological score was evaluated microscopically in a blinded manner. The membranous glomerulonephritis score (GN score) was evaluated using scoring from 0 to 4: 0, normal; 1, mild, focal or early proliferation; 2, moderate or definite proliferation and increased matrix; 3, diffuse and focal or diffuse proliferation; 4, severe diffuse proliferation with crescent/sclerosis. The renal interstitial nephritis score (IN score) was graded from 0 to 4 for inflammation and necrosis: 0, normal; 1, occasional, focal or small pockets of mononuclear cells (MNCs, 10–14 cells); 2, focal infiltration of MNCs (15–30 cells); 3, multifocal and extensive infiltration of MNCs; 4, severe infiltration of MNCs with extensive necrosis. Vasculitis was scored from 0 to 4: 0, normal; 1, occasional perivascular infiltration of MNCs; 2, several foci of perivascular infiltration of MNCs without necrosis; 3, multifocal perivascular infiltration of MNCs with/without necrosis; 4, multifocal or diffuse perivascular infiltration of MNCs; extensive with necrosis.

### Splenomegaly and lymphadenopathy

Spleen and cervical lymph nodes were removed and their weights were measured before fixation.

### Flow cytometry

A single cell suspension from the spleen was prepared by homogenizing the spleen through a 40-μm mesh nylon cell strainer (BD Falcon 352340, BD Biosciences, San Jose, CA, USA). Red blood cells were lysed using ACK lysing buffer (Gibco). Splenocytes were stained with fluorescein isothiocyanate (FITC)-conjugated rat anti-mouse CD45R/B220 (BD Biosciences), phycoerythrin (PE)-conjugated hamster anti-mouse CD69 (BD Biosciences), PE-conjugated rat anti-mouse GL7 (BD Biosciences), and PE-conjugated rat anti-mouse CD138 (BD Biosciences). At least 10,000 stained cells were counted and were analyzed with FACSCalibur™ flow cytometry (BD Biosciences). All splenocytes were primarily gated on live lymphocytes based on forward scatter (FCS) and side scatter (SSC). Germinal center B cells were identified as B220^+^ and GL7^+^. Activated B cells were identified as B220^+^ and CD69^+^, and plasma B cells were identified as B220^+^ and CD138^+^.

### Statistical analysis

Data are expressed as the mean ± SEM. Analysis of the significance of differences was performed using one-way analysis of variance (ANOVA) (for parametric data) or the Kruskal–Wallis test (for non-parametric data) using GraphPad Prism (version 5.0, La Jolla, CA, USA). In Kaplan-Meier survival curve, the curve comparison was resulted of the log-rank test using GraphPad Prism.

## Results

### HM71224 regulates BCR signaling in B cells and FcγR signaling in monocytes

**Fig. 1:**
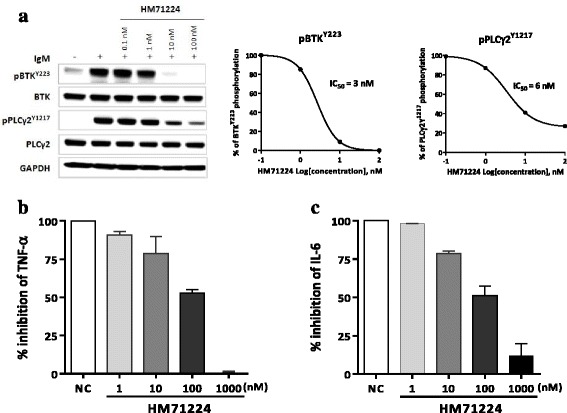
Inhibitory effects of HM71224 on B cell antigen receptor (BCR) and Fc receptor (FcR) signaling. **a** HM71224 dose-dependently inhibited BCR stimulation (1 μM of goat F(ab’)2 anti-human IgM)-induced phosphorylation of both Bruton’s tyrosine kinase (*BTK*) Y223 and phosphoinositide phospholipase C-γ2 (*PLCγ2*) Y1217 in Ramos cells. The western blots were reproduced three times and the representative one was chosen. **b**, **c** THP-1 cells (n = 2) were differentiated to macrophages by 10 ng/ml of interferon-γ. HM71224 treatment resulted in a trend toward reduction of IgG-induced TNF-α and IL-6 production by THP-1 cells. Data are shown as the mean ± SEM. *GAPH* glyceraldehyde 3-phosphate dehydrogenase

To characterize the inhibitory effect of HM71224 on the BCR signaling pathway, the activation of BTK and PLCγ2, the physiological substrate of BTK, following stimulation with anti-IgM was examined in human Ramos B lymphoma cells. HM71224 blocked both autophosphorylation of BTK and phosphorylation of PLCγ2 with IC_50_ values of less than 10 nM (Fig. 1a). To explore the inhibitory effect of HM71224 on the FcγR signaling cascade, the production of the inflammatory cytokines TNF-α and IL-6 was evaluated following stimulation with anti-IgG in human monocyte THP-1 cells. Production of both TNF-α and IL-6 effectively decreased in a dose-dependent manner (Fig. 1b and c).

### HM71224 inhibits B cell activation in animal models of lupus

**Fig. 2:**
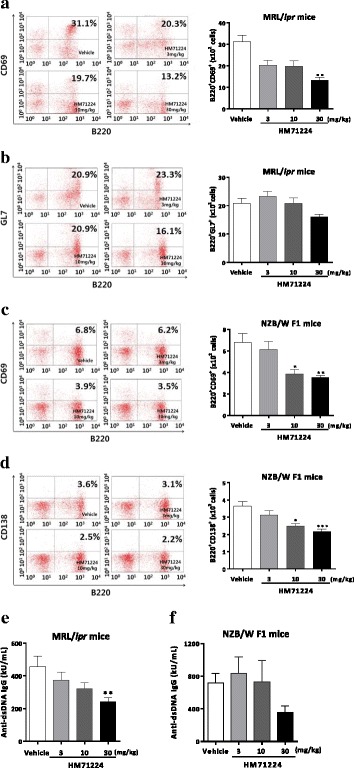
HM71224 inhibits B cells and autoantibody. Quantitative analysis of splenic B cell populations was performed by flow cytometry. HM71224 dose-dependently reduced activated B cells (**a**), germinal center B cells (**b**) and showed a significant ameliorative effect on activated B cells at 30 mg/kg after treatment for 20 weeks in MRL/*lpr* mice (*n* = 11 per vehicle-treated group, *n* = 12 per HM71224-treated group). Treatment with HM71224 for 24 weeks in NZB/W F1 mice resulted in a significant decrease in both activated (**c**) and plasma B cells (**d)** (*n* = 8 per vehicle-treated group, *n* = 9 per 3 mg/kg of HM71224-treated group, *n* = 12 per 10 and 30 mg/kg of HM71224-treated group). Serum anti-dsDNA IgG was measured by ELISA at the end of the study. HM71224 dose-dependently inhibited autoantibody in both mouse models (**e**, **f**). Data are shown as the mean ± SEM. *P < 0.05, **P < 0.01, and ***P < 0.001 versu*s* vehicle control, analyzed by the Kruskal–Wallis test

To determine whether HM71224 effectively inhibits B cells, splenic B cell subpopulations were evaluated using flow cytometric analysis. In MRL/*lpr* mice, the proportion of B220^+^CD69^+^ activated B cells dose-dependently decreased (*P* < 0.01 at 30 mg/kg, Fig. 2a) and that of B220^+^GL7^+^ germinal center B cells decreased without reaching statistical significance (Fig. 2b). In NZB/W F1 mice, both activated B cells and plasma B cells decreased in a dose-dependent manner. In addition, B220^+^CD69^+^ activated B cells (*P* < 0.01, Fig. 2c) and B220^+^CD138^+^ plasma B cells (*P* < 0.001, Fig. 2d) were significantly reduced at 30 mg/kg of HM71224 compared with those in vehicle-treated mice.

Moreover, HM71224 dose-dependently suppressed serum anti-dsDNA IgG in both MRL/*lpr* (*P* < 0.01 at 30 mg/kg, Fig. 2e) and NZB/W F1 mice (*P* not significant, Fig. 2f).

### HM71224 reduces spleen and lymph node enlargement

**Fig. 3:**
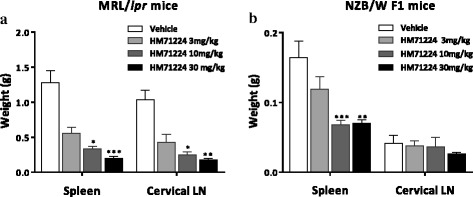
HM71224 inhibits enlargement of the spleen and lymph nodes resulting from disease development. **a** In MRL/*lpr* mice, the organ weights of the spleen and cervical lymph nodes (*LN*) were significantly reduced by treatment with 10 and 30 mg/kg of HM71224 for 20 weeks (*n* = 11 per vehicle-treated group, *n* = 12 per HM71224-treated group). **b** In NZB/W F1 mice, spleen weight was significantly reduced by HM71224 treatment, whereas the weight of cervical lymph nodes showed a nonsignificant trend toward decrease after treatment for 24 weeks (*n* = 8 per vehicle-treated group, *n* = 9 per 3 mg/kg of HM71224-treated group, *n* = 12 per 10 and 30 mg/kg of HM71224-treated group). Data are expressed as mean ± SEM. *P < 0.05, **P < 0.01, and ***P < 0.001 versus vehicle control, analyzed by the Kruskal–Wallis test

To investigate the inhibitory effects for splenomegaly and lymphadenopathy as manifestations of SLE, the weights of the spleen and lymph node were measured after 20 and 22 weeks of HM71224 treatment in MRL/*lpr* and NZB/W F1 mice, respectively. Spleen weights decreased (*P* < 0.001, Fig. 3a and b) in HM71224-treated MRL/*lpr* and NZB/W F1 mice, respectively. Cervical lymph node weight was reduced (*P* < 0.001, Fig. 3a and *P* = not significant, Fig. 3b) in HM71224-treated MRL/*lpr* and NZB/W F1 mice, respectively, compared with those of vehicle-treated mice.

### HM71224 prevents skin lesions

**Fig. 4:**
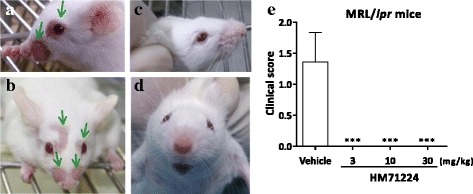
HM71224 prevents skin lesion progression. Skin lesions were observed in vehicle-treated mice around the nose or eyes (*arrows* show representative skin lesions) (**a**, **b**) compared with HM71224-treated MRL/*lpr* mice (**c**, **d**). **e** Skin lesion severity score was 1.4 ± 0.5 (mean ± SEM) in vehicle-treated mice, whereas no skin lesions developed in HM71224-treated MRL/*lpr* mice (*P* < 0.001) at 28 weeks of age (*n* = 11 per vehicle-treated group, *n* = 12 per HM71224-treated group)

To investigate the ameliorative effects of HM71224 for cutaneous lupus erythematosus, macroscopic skin lesions were observed in MRL/l*pr* mice with the development of spontaneous lupus-erythematosus-like skin lesions. Alopecia and scab formation were markedly developed in aged MRL/*lpr* mice. These typical skin lesions caused by progression of lupus were observed around the nose, ears, and intrascapular area of vehicle-treated mice (Fig. 4a and b), with lesion scores of 1.4 ± 0.5 (Fig. 4e), whereas no skin lesions developed in the mice treated with HM71224, irrespective of the dose levels, up to 28 weeks of age (Fig. 4c-e).

### HM71224 limits renal dysfunction in animal models of lupus

**Fig. 5:**
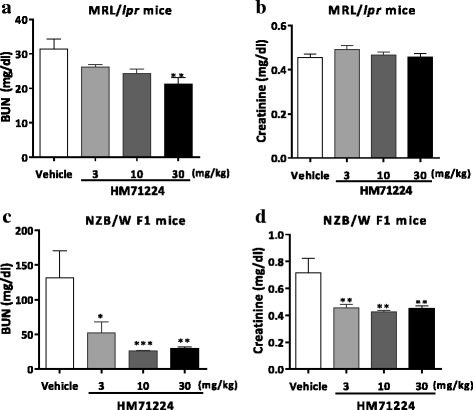
HM71224 limits renal impairment. Serum blood urea nitrogen (*BUN*) and creatinine (CRE) were measured at the end of the study. Treatment of MRL/*lpr* mice with HM71224 reduced BUN (**a**) but increased CRE was not observed at 28 weeks of age, even in vehicle-treated mice (**b**). Treatment of NZB/W F1 mice with HM71224 resulted in a significant decrease in BUN (**c**) and CRE (**d**) at 40 weeks of age. Data are shown as the mean ± SEM. *P < 0.05, **P < 0.01, and ***P < 0.001 versus vehicle control determined by analysis of variance (BUN and CRE in **c** and **d**) and the Kruskal–Wallis test (BUN in **a**)

**Fig. 6:**
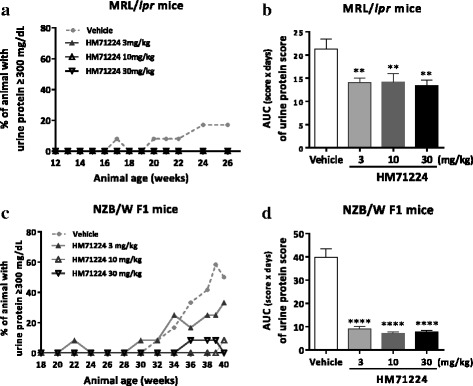
HM71224 reduced glomerular dysfunction in animal models of lupus. The severity of urine protein concentration was graded using a urine protein scoring system. The percentage of mice with severe proteinuria (urine protein ≥ 300 mg/dl or urine protein score ≥ 3) sequentially increased in both mouse models (**a**, **c**). Especially, severe proteinuria in NZB/W F1 mice dramatically increased from 30 weeks of age in vehicle-treated mice, whereas few HM71224-treated mice had severe proteinuria **c**. The area under the curve (*AUC*) for the urine protein score in all treatment periods was significantly reduced in HM71224-treated groups in both mouse models (**b**, **d**). Data are shown as the mean ± SEM. **P < 0.01 and ****P < 0.0001 versus vehicle control determined by analysis of variance

To determine the effects of HM71224 on renal impairment, we measured BUN and CRE at the end of the study, and to assess glomerular dysfunction, we measured urine protein once weekly or biweekly. Both serum BUN and CRE and urine protein increased more dramatically in vehicle-treated NZB/W F1 mice than in vehicle-treated MRL/*lpr* mice. Serum BUN was significantly ameliorated in both mouse models with HM71224 treatment, whereas serum CRE was significantly reduced in NZB/W F1 mice only (Fig. 5). The percentage of mice with severe proteinuria (urine protein ≥ 300 mg/dl or urine protein score ≥ 3) sequentially increased in both mouse models, and vehicle-treated NZB/W F1 mice especially had dramatically increased high-grade proteinuria, whereas HM71224 decreased the severe proteinuria in both mouse models (Fig. 6a and c). Although there was some fluctuation in the proteinuria score, the area under the curve (AUC) for proteinuria during the treatment periods was significantly decreased in HM71224-treated MRL/*lpr* and NZB/W F1 mice (Fig. 6b and d).

### HM71224 ameliorates renal injury and inflammation in animal models of lupus

**Fig. 7:**
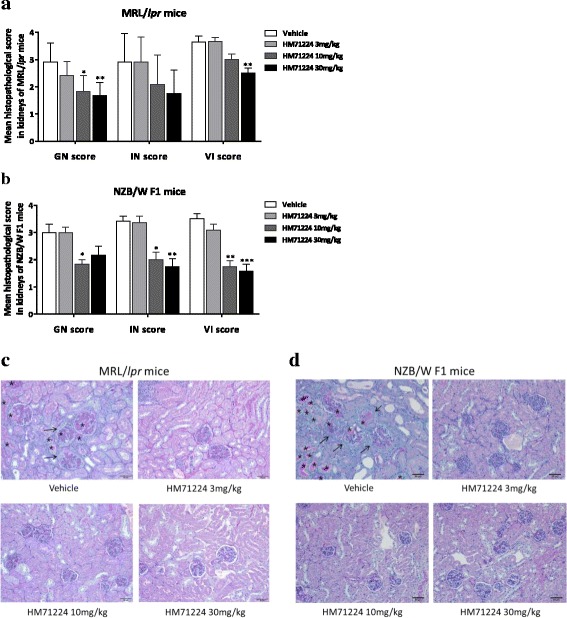
HM71224 ameliorates renal damage and inflammation in animal models of lupus. Renal histopathological analysis was performed using the membranous glomerulonephritis score (*GN*) score, the renal interstitial inflammation/fibrosis (*IN*), and the vasculitis (*VI*) score. **a** Treatment of MRL/*lpr* mice with HM71224 for 20 weeks produced a dose-dependent reduction in renal destruction and lymphocyte infiltration and a statistically significant reduction in GN and VI scores. **b** Treatment of NZB/W F1 mice with HM71224 for 22 weeks resulted in statistically significant amelioration of the GN, IN, and VI scores. Scores are shown as the mean ± SEM. *P < 0.05, **P < 0.01, and ***P < 0.001 versus vehicle control determined by the Kruskal–Wallis test. Representative histopathological Periodic acid Schiff stained sections of the kidneys of MRL/*lpr* mice at 28 weeks of age (**c**) and NZB/W F1 mice at 40 weeks of age (**d**). Thickening of the basement membrane (*arrow*), tubule distention with proteinaceous fluid (*asterisk*), and inflammation were ameliorated in the HM71224-treated groups. All images were captured at × 200 magnification

To assess whether HM71224 can limit renal damage and inflammation, histopathological analysis was conducted in both animal models. Among MRL/*lpr* mice, the vehicle-treated mice had moderate renal damage, as indicated by the membranous glomerulonephritis (GN) score (2.9 ± 0.7), renal interstitial inflammation (IN) score (2.9 ± 1.0), and vasculitis (VI) score (3.6 ± 0.2), whereas HM71224-treated mice had a dose-dependent decrease in the GN score (reduced by 43%, *P* < 0.01), IN score (reduced by 40%, *P* not significant), and VI score (reduced by 31%, *P* < 0.01) (Fig. 7a). Among NZB/W F1 mice, the vehicle-treated mice had moderate to severe renal damage, as indicated by the GN score (3.0 ± 0.3), IN score (3.4 ± 0.2), and VI score (3.5 ± 0.2), whereas HM71224-treated mice had significantly less renal damage and inflammation, as shown by a 39% reduction in GN score (*P* < 0.05), 49% reduction in IN score (*P* < 0.01), and 55% reduction in VI score (*P* < 0.001) (Fig. 7b). Moreover, HM71224 ameliorated the glomerular basement membrane thickening and tubule distention containing proteinaceous casts in the kidney in NZB/W F1 mice (Fig. 7c).

### HM71224 improves the survival rate in animal models of lupus

**Fig. 8:**
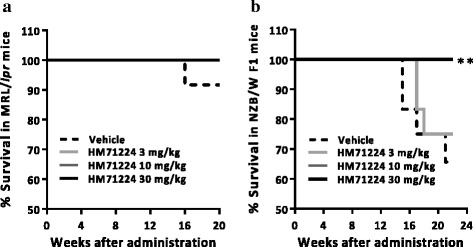
HM71224 increases the survival rate in animal models of lupus. **a** None of mice treated with HM71224 died through all by 20 weeks after administration compared to the survival rate of 92% (11/12) in vehicle treated MRL/*lpr* mice. **b** All mice treated with 10 and 30 mg/kg of HM71224 survived through all by 22 weeks following administration and had a statistically significantly better survival rate, whereas vehicle-treated mice and mice treated with 3 mg/kg of HM71224 had survival rates of 67% (8/12) and 75% (9/12), respectively, among NZB/W F1 mice (n = 12 per group). **P < 0.01 versus vehicle control determined by the log-rank test

Because the development of murine SLE-like disease features ultimately lead to premature death, the cumulative survival rate was estimated during treatment with HM71224. None of the MRL/*lpr* mice treated with HM71224 died during the course of treatment, whereas one of the vehicle-treated mice died at 16 weeks (Fig. 8a). Furthermore, in NZB/W F1 mice, the survival rate at 22 weeks in vehicle-treated mice was 67%, while all mice treated with 10 or 30 mg/kg of HM71224 were alive at 22 weeks (*P* < 0.01) (Fig. 8b).

## Discussion

B cell hyperactivation and immune complex-mediated FcR activation are known to play a major role in the pathogenesis of immune-mediated LN [5–9]. A previous report demonstrated the benefits of BTK inhibition in mouse models of TLR7/IFN-driven lupus by affecting both BCR and FcR signaling [23]. We also have previously demonstrated that BTK inhibition by HM71224 effectively blocks not only the phosphorylation of both BTK and PLCγ2 through downstream BCR signaling in human B cells, but also FcR-stimulated cytokine production in human monocytes [27].

Our present study demonstrates that HM71224 effectively reduced the numbers of several splenic B cell types. Germinal centers are central to development of long-lived plasma B cells, their clonal expansion, and somatic hypermutation affinity maturation. In SLE a large proportion of these autoreactive plasma cells, which are generated in the spleen, preferentially migrate to the inflamed kidneys. Therefore, the spontaneous formation of these germinal centers, which are an important source of autoreactive plasma cells, is considered a hallmark of SLE, and disease progression of LN is associated with an increase in the number and size of germinal centers [33, 34]. In this study, HM71224 dose-dependently and considerably decreased the number of splenic B220^+^GL7^+^ germinal center B cells, and this inhibition of B cell differentiation for germinal center formation may exert a therapeutic effect on LN. Inflamed tissue and secondary lymphoid organs can contribute to the number of autoreactive plasma cells in SLE [35]. In this study, HM71224 dose-dependently and significantly decreased the number of splenic B220^+^CD138^+^ plasma cells, which are considered the major producers of autoantibodies. Moreover, HM71224 strikingly inhibited splenic B220^+^CD69^+^ B cell numbers, which are early markers of B cell activation and antigen presentation. This result is consistent with previously reported data showing that B cells with defective BCR signaling fail to mature and are non-responsive to BCR cross-linking in terms of proliferation and upregulation of the activation marker CD69 [36, 37]. These results suggest that HM71224 may effectively inhibit autoantibody production and cytokine enhancement by B cells, which are caused by presenting autoantigens to CD4^+^ T cells as antigen-presenting cells and stimulating dendritic cells.

These numerous inhibitory effects of splenic B cells are consistent with the prevention of splenomegaly and enlargement of cervical lymph nodes observed following treatment with HM71224. Approximately 50% of patients with SLE have enlarged lymph nodes and these are usually detected in the cervical, axillary, and inguinal areas. In particular, lymphadenopathy is more frequently noted at the onset of disease or during disease flares [38]. Splenomegaly also occurs in 10–45% of patients with SLE owing to disordered immunoregulation and is particularly observed during active disease [39]. Thus, inhibition of both lymph node enlargement and splenomegaly are considered to be positive signals of the suppression of SLE-like disease progression by HM71224 in an animal model of lupus.

The first major therapeutic finding was that treatment with HM71224 resulted in no skin lesions in MRL/*lpr* mice. Skin rash is the second most common clinical manifestation in patients with SLE and in MRL/*lpr* mice, which represent a unique mice strain, with development of a skin rash [40]. The pathogenesis of skin lesions, hair loss, and scab formation in MRL/*lpr* mice is characterized by immunoglobulin and/or complement depositions and accumulation of various other components of the immune system including macrophage/monocytes and dendritic cells [41, 42]. The B cell deficient and spleen tyrosine kinase (SYK) inhibitor-treated MRL/*lpr* mice were found not to develop spontaneous dermatitis [8, 43] and rituximab has been shown to have a therapeutic effect against cutaneous lesions in patients with SLE [44]. Here, we demonstrated that the typical skin lesions around the nose or eyes were observed in vehicle-treated mice but not in HM71224-treated mice, and these effects in skin lesions may be associated with B cell modulation by HM71224.

The second major therapeutic finding was that HM71224 had therapeutic effects related to the amelioration of kidney damage caused by lupus-like renal inflammation. Intrarenal B cells can contribute to renal damage and inflammation by enhancing the immunological response as antigen-presenting cells, inducing cytokine-promoting T cell proliferation and lymphatic angiogenesis, and enhancing the local immune response to persisting autoantigens in the tubulointerstitium [45, 46]. In both MRL/lpr and NZB/W F1 mice, activation of TLR9 caused accelerated LN [47]. On the other hand, LN develops via unique mechanisms in each strain like the highly heterogenous nature of SLE. NZB/W F1 mice promote the renal damages B cell dependent manner including secretion of autoantibody, whereas MRL/lpr mice develop the renal damages via antigen presentation and cytokine production by B cells, not secretion of autoantibodies [5].

As discussed previously, HM71224 markedly reduced splenic B cell numbers, including those of germinal center B cells, plasma B cells, and activated B cells. We also reported the inhibitory effects of HM71224 on BCR and FcR signaling [27]. These mechanisms of action of HM71224 might lead to significant amelioration of renal injury and lymphocyte infiltration in both animal models, although differences in strains in the pathogenesis of LN can lead to different degrees of therapeutic drug efficacy. Therefore, we suggest that the therapeutic effects of HM71224 in glomerulonephritis, interstitial nephritis, and vessel inflammation in murine LN were mediated by B cell inhibition.

Finally, one of the major therapeutic findings was that there was no mortality during the period of HM71224 administration in both mice models (Fig. 8). Prevention of renal damage by HM71224 might have led to the improvement in survival rate, because LN leads to proteinuria and renal failure and is associated with significant mortality [48]. Although cyclophosphamide treatment has markedly reduced mortality in LN from more than 70% in the 1960s to less than 10% in recent years, mortality remains relatively high [49]. Therefore, despite continuous improvements in SLE therapeutics, mortality remains a serious problem in LN therapy [3, 4]. Thus, the most notable result of this study is that HM71224 can markedly increase the survival rate of animals with SLE by preventing renal damage and inflammation.

## Conclusions

This study indicated that HM71224 causes B cell modulation and has therapeutic effects via BTK inhibition of disease progression in SLE and LN in murine models of lupus.

## Abbreviations

ACK: Ammonium-chloride-potassium
ANOVA: Analysis of variance
AUC: Area under the curve
BCR: B cell antigen receptor
BMX: Bone-marrow tyrosine kinase
BTK: Bruton’s tyrosine kinase
BUN: Blood urea nitrogen
CO_2_: Carbon dioxide
CRE: Creatinine
dsDNA: Double strand deoxyribonucleic acid
ECL: Enhanced chemiluminescence
ELISA: Enzyme-linked immunosorbent assay
Fc: Crystallizable fragment
FcR: Crystallizable fragment receptor
FCS: Forward scatter
FITC: Fluorescein isothiocyanate
GAPH: Glyceraldehyde 3-phosphate dehydrogenase
GN: Glomerulonephritis
IC_50_: Half maximal inhibitory concentration
IFN-γ: Interferon gamma
IgG: Immunoglobulin G
IgM: Immunoglobulin M
IL-6: Interleukin 6
IN: Interstitial nephritis
LN: Lupus nephritis
MNC: Mononuclear cell
NZB/WF1: New Zealand Black/White F1
PE: phycoerythrin
PLCγ: Phosphoinositide phospholipase C gamma
RIPA: Radioimmunoprecipitation assay
SDS-PAGE: Sodium dodecyl sulfate polyacrylamide gel electrophoresis
SEM: Standard error of mean
SLE: Systemic lupus erythematosus
SSC: Side scatter
SYK: Spleen tyrosine kinase
TEC: Tec protein tyrosine kinase
TLR: Toll-like receptor
TNF-α: Tumor necrosis factor alpha
VI: Vasculitis
*xid*: X-linked immunodeficiency
